# Seagrass-rafted large benthic foraminifera transported into the deep Red Sea

**DOI:** 10.1038/s41598-025-90047-7

**Published:** 2025-02-17

**Authors:** Marleen Stuhr, Hildegard Westphal, Fabio Marchese, Guillem Mateu-Vicens, Francesca Giovenzana, Thomas Lüdmann, Volker Vahrenkamp, Marco Taviani

**Affiliations:** 1 Leibniz Centre for Tropical Marine Research (ZMT), Bremen, Germany; 2 Department of Geosciences, Bremen University, Bremen, Germany; 3 Physical Sciences and Engineering Division, King Abdullah University of Science and Technology (KAUST), Thuwal, Saudi Arabia; 4 Biological and Environmental Sciences and Engineering Division, King Abdullah University of Science and Technology (KAUST), Thuwal, Saudi Arabia; 5 Department of Biology, Universitat de Les Illes Balears, 07122 Palma de Mallorca, Spain; 6 Institute of Geology, University of Hamburg, Hamburg, Germany; 7 ISMAR-CNR, Bologna, Italy; 8 Stazione Zoologica ‘Anton Dohrn’, Napoli, Italy

**Keywords:** Benthic foraminifera transport, Seagrass epiphytes, Deep-sea sediments, Brine pool, Pelagic carbonates, Red Sea, Palaeontology, Sedimentology, Ecology

## Abstract

Large shallow-marine foraminifera tests occur in deep-sea carbonate sediments of the northern Red Sea as a minor but recurring component among the remains of otherwise pelagic and deep-marine benthic biogenic assemblages. In this study of sediments recovered along the northern shore of Saudi Arabia, the symbiont-bearing taxa *Sorites variabilis*, *S. orbiculus*, *Amphisorus hemprichii*, *Amphistegina lobifera*, *A. lessonii* and *A. radiata* were identified in samples from between 430 to 1,000 m depth. These foraminifera are dwelling in shallow-water environments, associated with coral reefs and seagrass habitats. The seemingly erratic occurrence of photosymbiotic benthic organisms in deep-sea sediments was explained by the finding of such foraminifera tests along with seagrass (e.g., *Halophila* leaves) and macroalgae remains in pristine preservational states in the sediment of the Umluj brine pool below ~ 638 m depth. This indicates a passive transport process by rafting attached to floating macrophytes to these off-platform settings. The abundant seagrass and oceanographic conditions along the Arabian Peninsula may facilitate the transport of epiphytes and associated taxa offshore. Such long-distance transport mechanisms could further contribute to the rapid (co-)dispersal of some of these organisms into new habitats. Passive rafting should thus be considered in interpretation of sedimentary records and biogeographic patterns.

## Introduction

Large benthic foraminifera (LBF) typically inhabit shallow, photic environments such as coral reefs in the (sub)tropics, where their photosymbiotic relationship makes them key contributors to carbonate production^1^. Their environmentally sensitive depth distribution usually makes them powerful proxies for palaeoecological reconstructions^2^, but their presence in deeper marine settings, well below the photic zone, raises questions about potential transport pathways.

The Red Sea, a narrow semi-enclosed basin at tropical-subtropical latitudes in arid climate conditions, provides a unique setting to investigate marine carbonate sedimentation processes under mesophotic to aphotic conditions near a coastal area with no to minimal riverine input. Limited water exchange with the Indian Ocean through the Bab el Mandeb Strait, combined with high evaporation rates, results in elevated salinities and minimal surface water renewal^3–5^. Seasonal temperature variations in the surface waters range from 26 °C in the north to 30 °C in the south during summer, and 20 to 26 °C during winter^6^. Wind-driven surface water circulation generates eddies and sub-gyres, influencing water column dynamics^7,8^. Regarding primary productivity, the Red Sea is classified as a prevalently oligotrophic basin^9^. The general physiography of this narrow basin is relatively simple, consisting of a wide main trough progressively deepening basinwards, with bathyal depths more than 2,500 m reached in its axial parts^10^. However, the morphology of the basin is highly heterogeneous with tectonic features such as pull-apart basins and ridges, volcanic mounds, and pronounced features caused by salt tectonics^11–13^. Altogether, such characteristics strongly influence the sedimentary processes from coastal to bathyal depths. For instance, one contrasting trait of the Quaternary Red Sea marine sedimentation regards the prolific production of carbonates through flourishing coral reef dominated factories in the euphotic zone^14^, to the upper mesophotic zone^15,16^, *versus* a minimal production by benthic factories below the photic zone, strongly controlled by highly demanding hydrological conditions. Primary production in the water column partially compensates this scarce deep-marine benthic carbonate production by adding skeletal components through pelagic calcifiers (nannoplankton, planktonic foraminifera and molluscs), with minor addition through nekton. The current regional aridity, with very limited riverine transport processes, prevents the input of substantial terrigenous components to marine sediments. The net result is that the deep-water standard sediment of the Red Sea is prevalently a mixture of > 90% of pelagic carbonates (nanno-globigerinid-pteropod carbonate ooze composed primarily of coccoliths, planktonic foraminifera, and pteropod shells) with subordinate terrigenous particles and minimal additions from benthic carbonate factories^17,18^. The scarce benthic carbonate coarse fraction (> 1 mm) mainly comprises of benthic bivalves, gastropods, echinoids, serpulids, and decapods. Fish otoliths are another minor carbonate component from the nekton. Consequently, and in the absence of gravitational sedimentation pathways in the rugged bathymetry, it is unexpected to find shallow-water benthic foraminifera in these deposits.

**Fig. 1:**
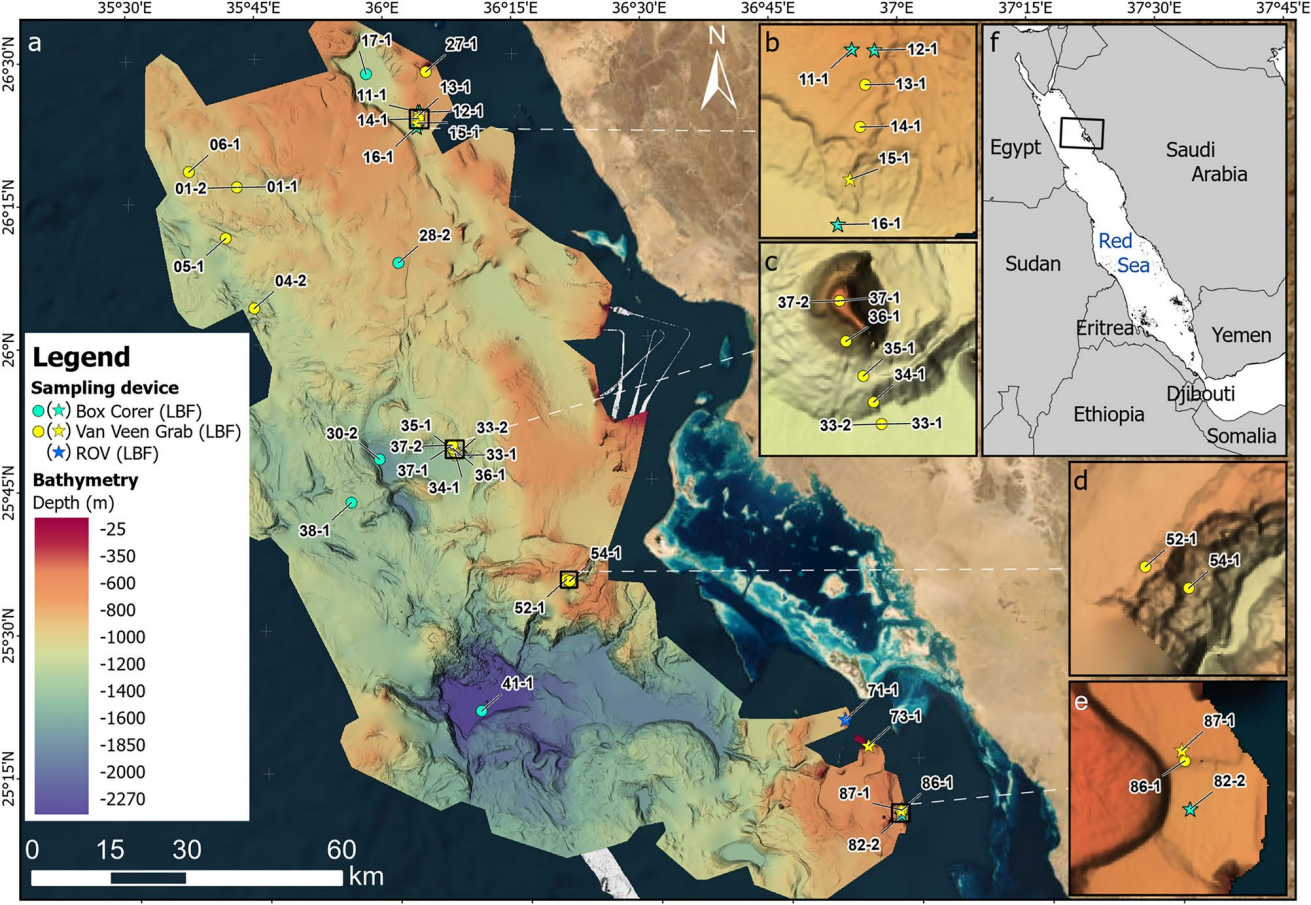
(**a**) Bathymetric map of the study area in the northeastern Red Sea offshore Al Wajh coastline, indicating sites of sample collection by giant box corer (green), van Veen grab (yellow), and remotely operated vehicle (blue), with close-ups showing subsections of the sampling area in detail (**b**–**e**) and the location of the study area within the Red Sea (**f**). Sampling sites at which shallow-dwelling benthic foraminifera were found in deep-sea sediments are indicated by stars instead of dots. The map was created using Esri ArcGIS® Pro software v3.4.0. (ESRI, 2024; World Imagery basemap credits: Esri, DigitalGlobe, GeoEye, i-cubed, USDA FSA, USGS, AEX, Getmapping, Aerogrid, IGN, IGP, swisstopo, and the GIS User Community).

In September 2023, cruise M-193 aboard RV *Meteor* surveyed a Saudi Arabian sector of the northern Red Sea^19^. The area explored encompasses a wide range of habitats, from upper mesophotic depths (~ 35 m) to deep-sea areas and basins at up to 2,250 m depth. It includes a deep-sea fan to the north, several isolated mini-basins related to salt tectonics, mesophotic coral bioherms, and a recently discovered brine pool system in the south (Fig. 1). A nearly continuous coral reef system and the Al Wajh carbonate platform characterize the coastal waters facing the study area.

One of the primary foci of the mission was the evaluation of marine carbonate genesis and deposition under mesophotic to aphotic conditions. For this purpose, some 60 bottom sediment samples were collected by means of a giant box corer and a van Veen grab sampler. This information was integrated by visual imaging and samples obtained by a remotely operated vehicle (ROV).

The analysis of bottom sediments from depths ~ 400 to 1000 m depth consistently disclosed the presence of photosymbiotic LBF in the sand-sized sediment fraction > 1 mm. The conspicuousness of these minor components was enhanced by the relative paucity of other benthic carbonate components in these otherwise strongly pelagic-dominated assemblages. The occurrence of photosymbiotic shallow-water foraminifera in aphotic deep-sea settings indicates a transport mechanism capable of moving these organisms far offshore. Therefore, this unexpected discovery prompted a more in-depth evaluation of their taxonomy and potential transport processes. Here we propose passive rafting on floating macrophytes as plausible vector for their displacement outside their habitats.

This case study closes the link between erratic occurrences of LBF in pelagic sediments by documenting the LBF taxa identified in these deep-sea sediments and, made possible by unique preservation in a brine pool, assessing the role of seagrass and macroalgae remains as transport agents. We also discuss the potential implications for sedimentary processes and dispersal mechanisms. By clarifying the source-to-sink pathway for these shallow-water organisms, we aim at improving our understanding of the distribution of LBF and other (facultative) epiphytes, as well as their occurrence in deep-sea carbonate settings.

### Study area

The study area is located along the eastern Red Sea margin off Saudi Arabia (between 25°50′33.99"N 36°25′29.47"E and 25°20′0.18"N 36°38′59.69"E, Fig. 1), offshore one of the largest shallow-water reef complexes in the Northern Red Sea, the Al Wajh carbonate platform^11^. It extends over ~ 200 km North to South, from 60–80 km northward of the northern end of Al Wajh carbonate platform to ~ 30 km southward, transitioning East to West over ~ 80 km from the shallow continental shelf into deeper waters of the Red Sea basin. The region is shaped by fault blocks, salt diapirs, and a siliciclastic delta^20^. Salt mobilisation processes, such as diapirism, have significantly influenced the submarine topography, resulting in the present complex seafloor morphology and has created small basins, ridges, and local highs^12,13,21^, and salt tectonics are also thought to have led to the disintegration of the southwestern Al Wajh platform^22^.

**Fig. 2:**
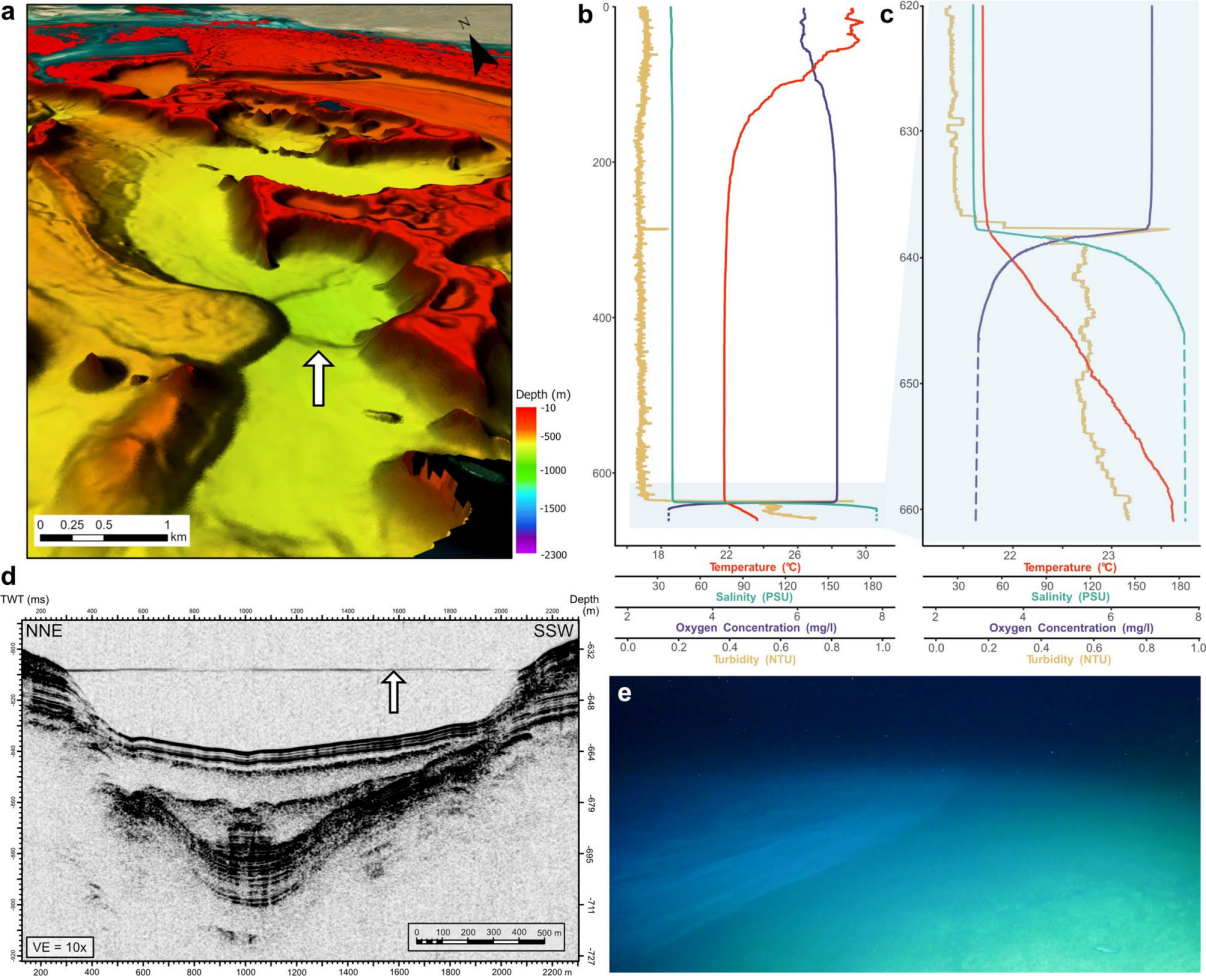
Umluj brine pool: (**a**) bathymetry around the brine pool (indicated by an arrow) and towards the coastline; (**b**) CTD cast profile of the water column above and inside the brine pool (station M193-82–1) showing seawater temperature, salinity, dissolved oxygen concentration, and turbidity from 2.5 to 661 m water depth with a pronounced increase in salinity and turbidity at around 638 m, below which oxygen concentration drops and temperature gradually increases, as shown in (**c**) a close-up of the water column between 620 and 661 m depth, with dashed lines indicating salinity and oxygen saturation values outside the instrumental range; (**d**) PARASOUND (parametric sub-bottom acoustic) profile showing the pycnocline as a single indistinct reflector (horizontal line) above the depression (indicated by an arrow); and (**e**) ROV image of the Umluj brine pool ‘beach’ area (station M193-85, dive depth 632 m). The pycnocline between marine waters above and the highly saline brine below is forming waves. Field of view along the bottom of the image is ~2.5 m.

A recently discovered feature of the study area is the Umluj brine pool, nested in a depression south of the platform (Fig. 1e; Fig. 2). This brine pool is characterised by extreme salinity (> 180 PSU), elevated temperatures (> 23 °C), low pH (< 6.5), anoxic conditions, and sulphur-rich waters (Petrovic et al., 2024^23^, this paper, Fig. 2). The strongly developed pycnocline is visible in our parametric sub-bottom acoustic (PARASOUND) profile (Fig. 2d,e).

The coastal waters are known to host a diverse array of ecosystems that contribute to sediment production and distribution, including extensive coral reefs, seagrass beds, and mangroves^24^. Coral reef systems play a dominant role in local biogenic carbonate production. The central lagoon of the Al Wajh carbonate platform, enclosed by a 115 km reef-shoal belt, also hosts dense seagrass beds and tidal flats, and contains poorly sorted, sand-sized sediments with notable lateral variability in carbonate fines^11,25^. The strong tidal currents passing through narrow channels, despite tidal amplitudes of less than 1 m, facilitate sediment resuspension and redistribution^26^, while hydrodynamic forces, such as the eastern boundary current, coupled with local wind-driven currents and mesoscale eddies further drive the transport of coastal biogenic carbonate, organic matter, and suspended sediment fines toward deeper areas^8,27,28^.

## Material and methods

The research cruise M193 was conducted over five weeks in September–October 2023 aboard the German RV *Meteor*^19^. A primary objective of the cruise was to investigate marine carbonate sedimentation in the Saudi Arabian Northern Red Sea, particularly in the vicinity of the Al Wajh carbonate platform, and was complemented by sea-floor mapping, Conductivity-Temperature-Depth (CTD) casts, and Remotely Operated Vehicle (ROV)-facilitated surveys.

Seafloor mapping was conducted using multibeam sonar systems (Kongsberg EM122 and EM710) and a parametric sub-bottom acoustic profiler system (Teledyne PARASOUND), collecting data at speeds of 5.5–6 knots or 7–9 knots depending on operational constraints. The multibeam sonar dataset was processed following the established workflow in QPS QimeraTM v2.6 (QPS Hydrographic and Marine Software Solutions, 2016) to generate a Digital Elevation Model (DEM) at a 30 m resolution for the surveyed area. Sound speed profiles for calibrating both multibeam systems were obtained from CTD profiles and processed with Sound Speed Manager^29^. The resulting map was created using Esri ArcGIS® Pro software v3.4.0. (Environmental Systems Research Institute (ESRI), 2024, Redlands).

CTD casts were performed to compile water column profiles at all major sampling stations, using the on-board SEA-BIRD 911 + CTD, equipped with a pressure sensor, conductivity and temperature sensors, dissolved oxygen sensors, altimeter, and FLNTUR (for chlorophyll a concentration and turbidity). The maintenance and calibration of all sensors were carried out by SEA-BIRD Kempten prior to the cruise. Data were processed using SBE Data Processing Version 5.30a and visualised with Ocean Data View Version 4.3.6^30^ and R Studio Version 2023.12.0 + 369^31^. Because instrumental calibrations and established conversions rely on ‘normal’ open ocean conditions, in-situ oxygen saturations were calculated based on these assumptions.

MARUM-Squid, a light work-class ROV targeted the brine pool surface and surrounding areas to assess the extent of this environment and its faunal characteristics (dive M193-85, Fig. 2e). The transect also covered areas of the pool ‘beach’, i.e., where the internal wave of the pycnocline between normal marine waters and the highly saline brine meet the seafloor. On ROV dive M193-71 (Fig. 1), a crust-like bulk surface sediment hash was collected.

Sediment sampling was conducted using a van Veen grab sampler at 51 stations (36–1,459 m depth) and a box corer at 12 stations (585–2,254 m depth). The samples studied here were taken from the surfaces, representing the seafloor, and bulk samples from the grabs and box cores (Fig. 1). All sediment samples were wet-sieved using 4 mm, 2 mm, and 1 mm mesh-size sieves, dried at 50 °C, and described for biogenic components. The box core recovered from the brine pool (M193-82–2) and the grab sample from the beach of the brine pool (M193-86–1) were additionally sampled for organic remains. These were carefully removed during sieving and subsequently air-dried in sampling dishes at room temperature, protected from light, heat or humidity.

Following the cruise, foraminifera tests and macrophyte components were documented at the Leibniz Centre for Tropical Marine Research (ZMT) in Bremen, Germany, using a Keyence VHX-5000 digital microscope. The taxonomic analysis of the benthic foraminifera, based on morphological characteristics, was conducted following the methodologies outlined by Hottinger et al. (1993)^32^, Lee et al. (2004)^33^ and Merkado et al. (2013)^34^. Nomenclature adheres to the standards established by the World Register of Marine Species^35^.

## Results

### Seafloor morphology

The seafloor bathymetry of the study region, based on the EM122 and EM710 datasets, was created at a resolution of 40 m, covering depths from 30 to 2200 m across an area of 9800 km^2^ (Fig. 1a). The northern sector of the study area features a steep continental slope descending from to 90–110 m to 900 m, followed by a gentler slope to 1100 m. This area is characterised by ridges, small basins, and morphologies related to salt tectonics, such as depressions, ridges, and mass-wasting deposits. The central sector, influenced by the Zabargad Fracture Zone, features a complex canyon system extending from the Al Wajh platform to a depth of 1770 m, with a small plateau accommodating canyon sediments. The southern sector resembles the northern sector with two basins at 670 m and 1300 m. The shallower basin near the Al Wajh platform contains drowned reefs and a brine pool below ~ 638 m (Fig. 1e, Fig. 2a), while the deeper basin shows continuous reflectors interrupted by disturbances, indicating hemipelagic sedimentation events.

The Umluj brine pool area at ~ 15 km south of the Al Wajh platform was surveyed by hydroacoustic surveys and PARASOUND profiles. Sharp changes in water physicochemical parameters in the uppermost meters of the brine cause a single horizontal reflection across the depression in the PARASOUND profile (Fig. 2d).

### Water column

The physical variables of the water column measured in the CTD casts exhibited general patterns with slightly increased salinity (from 39.39 PSU near the surface to ~ 40.5 below 180 m depth), decreasing temperature (from > 29 °C to 21.68 °C at ~ 400 m and below), while oxygen saturation increased from 6.15 mg/l (referring to ~ 188 µmol/kg) near the surface to 6.9 mg/l (~ 210 µmol/kg) below 450 m depth; Fig. 2b).

The CTD profile at the brine pool site (station M193-82–1) showed distinct layers in the water column, with a turbid layer on top of the brine surface (Fig. 2e) at ~ 638 m depth, followed by steep gradients inside the upper meters of the pool characterising significant changes in seawater density, salinity, and dissolved oxygen concentration (Fig. 2c). Water temperature increased gradually up to 23.5 °C within the brine, while most other variables were exceeding instrumental calibration and measurement ranges by far below ~ 645 m depth. The brine pool water exhibited extreme salinity of > 180 PSU, along with high seawater density (~ 1033.4 kg/m^3^ at 637.7 m depth and > 1146.1 kg/m^3^ below 646 m depth). Dissolved oxygen concentrations dropped at the surface of the brine pool, from 1.2 mg/l at 632 m depth to anoxic conditions (< 0.5 mg/l dissolved oxygen) at 637 m, and values around zero at 638 m, reaching instrumental limits. This refers to a drop from ~ 209 µmol/kg above to < 6 µmol/kg inside the brine, assuming normal seawater density and open ocean conditions. Water collected from the brine pool had a strong sulphur smell and viscous appearance.

### Shallow-water benthic foraminifera

We identified 29 specimens of photosymbiont-bearing benthic foraminifera in the > 1 mm fraction in seven grab or box core samples, as well as in one ROV-collected sample (Table 1), in samples from all 31 deep-water stations (Fig. 1). The distribution is limited to depths less than ~ 1,000 m, as no LBF have been discovered in deeper water samples, recovered from down to ~ 2,250 m depth.

**Table 1:** Shallow-water benthic foraminifera contained in deep marine samples, from North to South, including station number, depth, coordinates, species names and test conditions, and type of sediment corer used (BC = box corer, GRAB = van Veen grab sampler, ROV = remotely operated vehicle).

Station	Latitude	Longitude	Depth	Foraminifera species	Test condition	Corer
*11–1*	26°24,137’N	036°03,923’E	697 m	*Amphistegina lobifera*	Nearly pristine	BC
*11–1*	26°24,137’N	036°03,923’E	697 m	*Sorites orbiculus*	Slightly broken	BC
*12–1*	26°24,125’N	036°04,150’E	684 m	*Sorites orbiculus*	Pristine (broken)	BC
*12–1*	26°24,125’N	036°04,150’E	684 m	*Sorites orbiculus*	Slightly broken	BC
*12–1*	26°24,125’N	036°04,150’E	684 m	*Sorites variabilis*	Slightly broken	BC
*12–1*	26°24,125’N	036°04,150’E	684 m	*Sorites variabilis*	Slightly broken	BC
*12–1*	26°24,125’N	036°04,150’E	684 m	*Sorites variabilis*	Slightly abraded	BC
*12–1*	26°24,125’N	036°04,150’E	684 m	*Sorites variabilis*	Slightly abraded	BC
*15–1*	26°22,960’N	036°03,875’E	868 m	*Sorites orbiculus*	Slightly broken	GRAB
*16–1*	26°22,553’N	036°03,742’E	938 m	*Sorites orbiculus*	Nearly pristine	GRAB
*16–1*	26°22,553’N	036°03,742’E	938 m	*Sorites orbiculus*	Slightly abraded	GRAB
*16–1*	26°22,553’N	036°03,742’E	938 m	*Sorites orbiculus*	Slightly abraded	GRAB
*16–1*	26°22,553’N	036°03,742’E	938 m	*Sorites orbiculus*	Slightly abraded	GRAB
*16–1*	26°22,553’N	036°03,742’E	938 m	*Sorites orbiculus*	Slightly abraded	GRAB
*71–1*	25°19,241’N	036°51,624’E	441 m	*Amphistegina lessonii*	Strongly abraded	ROV
*71–1*	25°19,241’N	036°51,624’E	441 m	*Amphistegina radiata*	Slightly abraded	ROV
*71–1*	25°19,241’N	036°51,624’E	441 m	*Amphistegina radiata*	Strongly abraded	ROV
*73–1*	25°16,426’N	036°54,198’E	429 m	*Amphisorus hemprichii*	Nearly pristine	GRAB
*73–1*	25°16,426’N	036°54,198’E	429 m	*Amphisorus hemprichii*	Nearly pristine	GRAB
*73–1*	25°16,426’N	036°54,198’E	429 m	*Amphisorus hemprichii*	Nearly pristine	GRAB
*82–2*	25°09,089’N	036°57,800’E	668 m	*Sorites orbiculus*	Pristine (broken)	BC
*82–2*	25°09,089’N	036°57,800’E	668 m	*Sorites orbiculus*	Pristine (broken)	BC
*82–2*	25°09,089’N	036°57,800’E	668 m	*Sorites orbiculus*	Nearly pristine	BC
*82–2*	25°09,089’N	036°57,800’E	668 m	*Sorites orbiculus*	Nearly pristine	BC
*82–2*	25°09,089’N	036°57,800’E	668 m	*Sorites variabilis*	Nearly pristine	BC
*82–2*	25°09,089’N	036°57,800’E	668 m	*Sorites variabilis*	Slightly abraded	BC
*82–2*	25°09,089’N	036°57,800’E	668 m	*Sorites variabilis*	Strongly abraded	BC
*87–1*	25°09,596’N	036°57,737’E	632 m	*Amphisorus hemprichii*	Nearly pristine	GRAB
*87–1*	25°09,596’N	036°57,737’E	632 m	*Amphisorus hemprichii*	Slightly abraded	GRAB

Station 82-2 is within the Umluj brine pool and station 87-1 is on the beach of the brine pool.

**Fig. 3:**
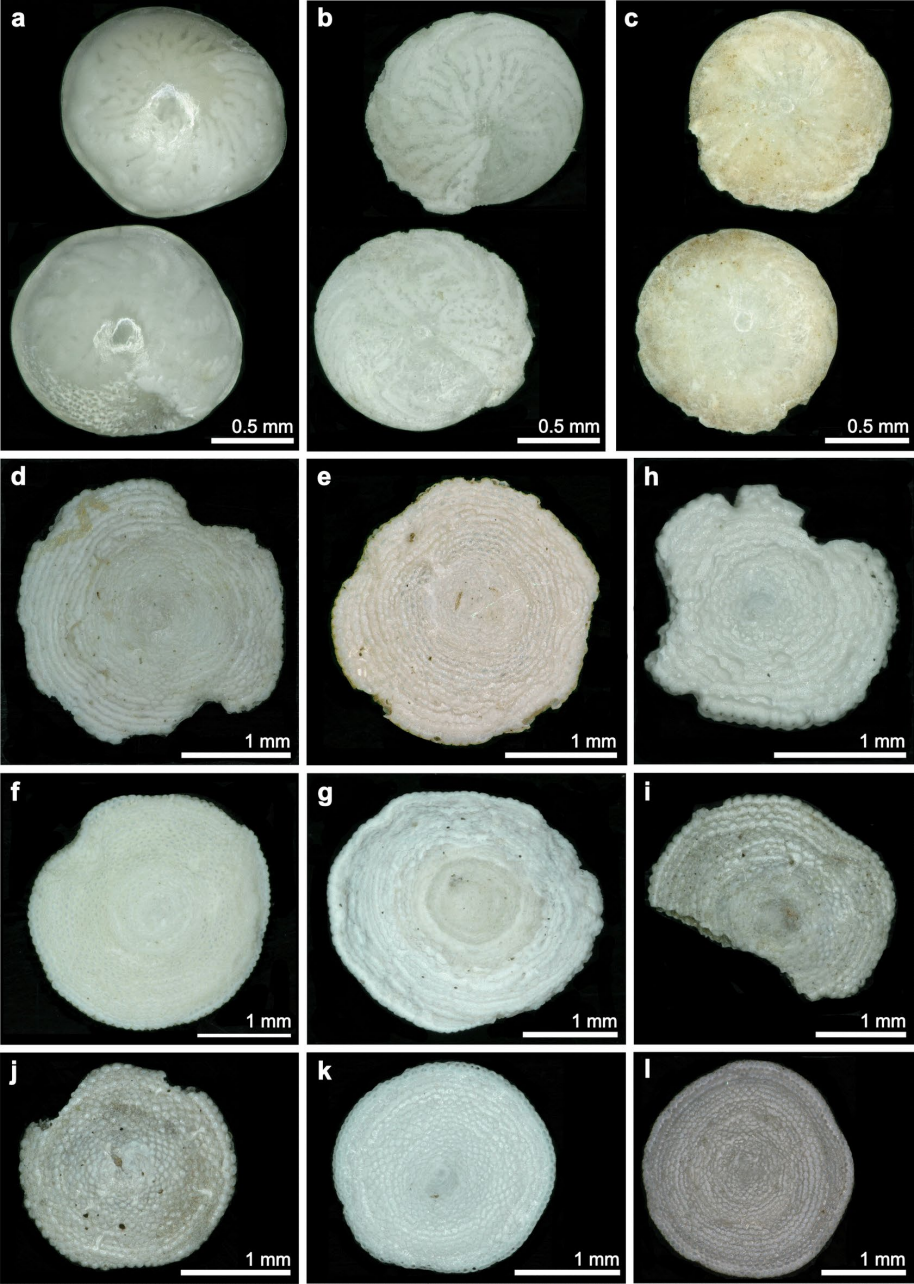
Spiral (upper) and umbilical views (lower image) of (**a**) *Amphistegina lobifera*, station 11; (**b**) *A. radiata*, station 71; (**c**) *A. lessonii*, station 71; and spiral views of (**d**) *Sorites variabilis*, station 12 and (**e**) station 82; (**f**) *Amphisorus hemprichii*, station 73 and (**g**) station 87; (**h**) *Sorites orbiculus*, station 11, (**i**) station 12, (**j**) station 15, (**k**) station 16, and (**l**) station 82. Lateral views are shown in the supplementary materials Figure S1.

**Fig. 4:**
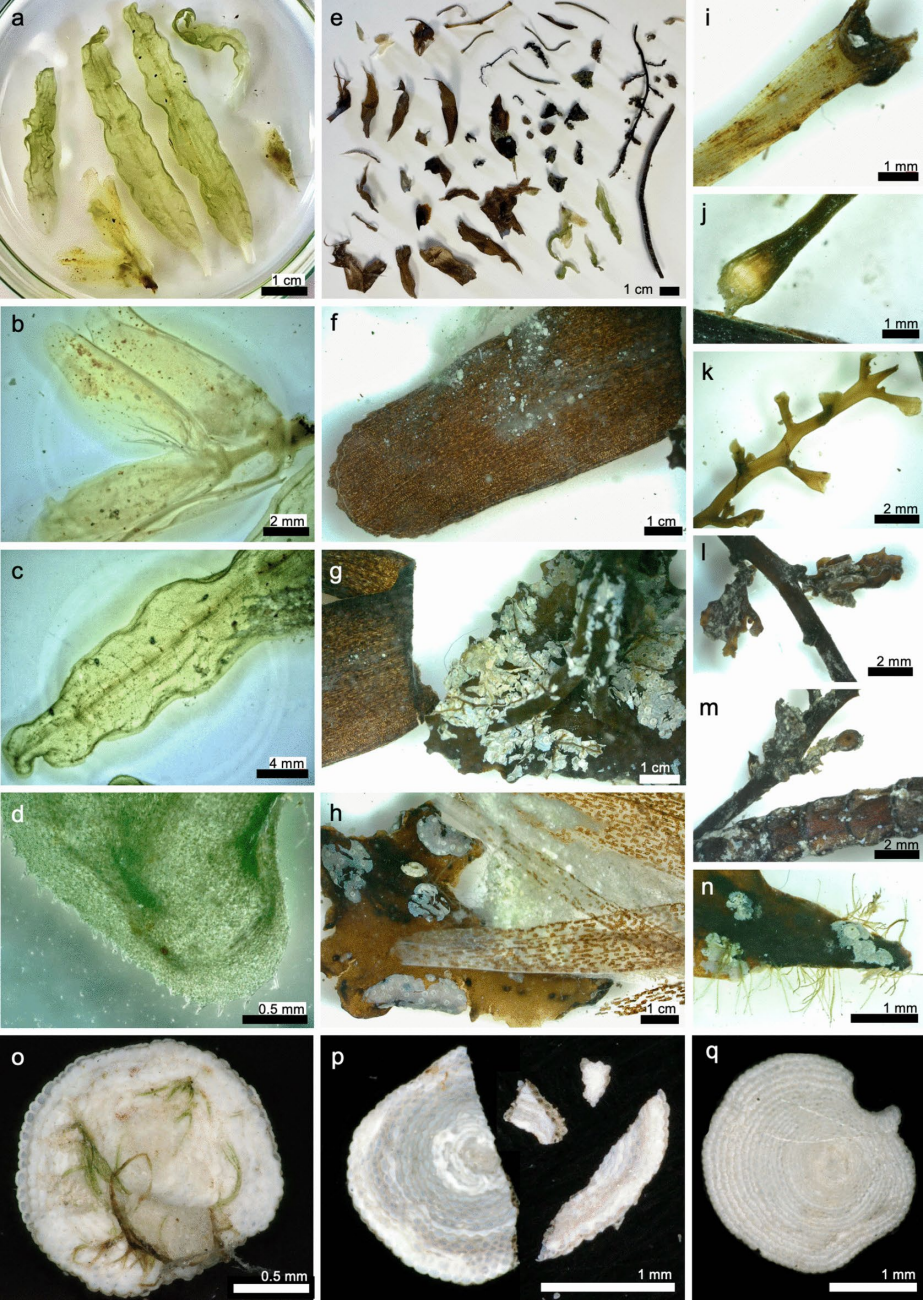
Macrophytes, algal remains and large benthic foraminifera from the box core retrieved from the Umluj brine pool (M-193–82-2): (**a**–**d**) (parts of) seagrass leaves, sheaths, a stem, and rhizomes resembling *Halophila stipulacea* or *H. ovalis*; (**e**) overview of all macrophyte remains found in the sample; (**f**–**h**) close-ups of other seagrass remains originating from *Thalassodendron ciliatum* or *Cymodocea serrulata*; (**i**–**n**) parts of other macrophytes, such as brown algae (*Turbinaria ornata*, (**g**,**n**)), small parts of *Sargassum* spp. (**l**,**m**), and red algae (*Laurencia* spp., (**k**)), were identified partly covered in calcifying epibionts; (**o**–**q**) *Sorites* spp. specimens with small remains or imprints of their phytal substrates ((**o**,**p**) = *S. orbiculus*, (**q**) = *S. variabilis*). Further Soritidae from the brine pool bottom and beach are shown in Fig. 3e,g,l. Their appearance is generally pristine, as (**p**) test was preserved well and later broke during handling, highlighting the delicate nature of these carbonate components, which points to a gentle way of transport such as rafting.

The most commonly encountered species was *Sorites orbiculus* Forsskål in Niebuhr, 1775 (n = 13 at four stations), followed by *S. variabilis* Lacroix, 1940 (n = 7 at two stations), *Amphisorus hemprichii* Ehrenberg, 1838 (n = 5 at two stations), *Amphistegina radiata* Fichtel and Moll, 1798 (n = 2 at one station), and single specimens of *Amphistegina lessonii* d’Orbigny in Deshayes 1830 and *Amphistegina lobifera* Larsen, 1976 at separate stations (Table 1, Fig. 3, Fig. S1). Test preservation was generally high, with little or no breakage. Only the *A. lessonii* and *A. radiata* specimens from station 71 exhibited significant signs of abrasion (Fig. 3b,c, Fig. S1b,c). The box core from the brine pool (station M193-82) contained several *Sorites* spp. specimens (Figs. 3e,l, 4o–q, Table 1), with some still attached to the macrophyte remains. Nearly all tests from this sample were pristine, and one still showed colouration typical of living specimens. This fine test broke during handling, highlighting the brittle nature of these carbonate components.

Notably, well-preserved macrophyte remains were identified in the brine pool samples (M193-82-2, M196-86-1; Fig. 4). These included the roots and leaves of *Halophila stipulacea* (Forsskål) Ascherson, 1867, with distinctly serrated, elongated paddle-shaped leaves, and large transparent leaf sheaths (Fig. 4a–d). Parts of *Thalassodendron ciliatum* (Forsskål) Hartog 1970, recognisable by its long cylindrical leaf blades with serrated margins and rounded leaf tips, were also found (Fig. 4e–h). Additionally, fragments of wiry seagrass stems with leaf scars and wide linear leaves, which might belong to *Thalassodendron ciliatum* or *Oceana serrulata* (R. Brown) Byng & Christenh (previously *Cymodocea serrulata* (R. Brown) Ascherson & Magnus, 1870), were present (Fig. 4e,m). In addition to these angiosperms, parts of other macrophytes, such as brown algae (*Turbinaria ornata* (Turner) J. Agardh, 1848), small parts of *Sargassum* spp. C. Agardh, 1820, and red algae (*Laurencia* spp. J. V. Lamouroux, 1813), were identified (Fig. 4i–n). These macrophyte remains were in many cases covered with remnants of epibenthic encrusters such as tube building-worms or polychaetes, and bryozoans (e.g., see Fig. 4g–n), which were later detached during sample processing.

## Discussion

The discovery of photosymbiotic shallow-water LBF in deep-sea sediments at depths ranging between 400 and 1000 m in the Red Sea, suggests a transport mechanism other than periplatform sedimentation where these settings are sedimentologically disconnected from euphotic carbonate factories. This is the case especially in locations with otherwise exclusively typical pelagic components and an absence of gravity flows. The spatial distribution of samples containing these benthic taxa below the mesophotic zone from the northern to the southern limit of the study area, corresponds to a distance from the coastal shoreline or the shallow carbonate systems of the Al Wajh platform or other islands of up to 25 km (Fig. 1). The excellent preservation of these “exotic” specimens, particularly the delicate and fragile Soritidae shells, suggests minimal degradation or modification caused by sedimentary processes or water movement.

**Fig. 5:**
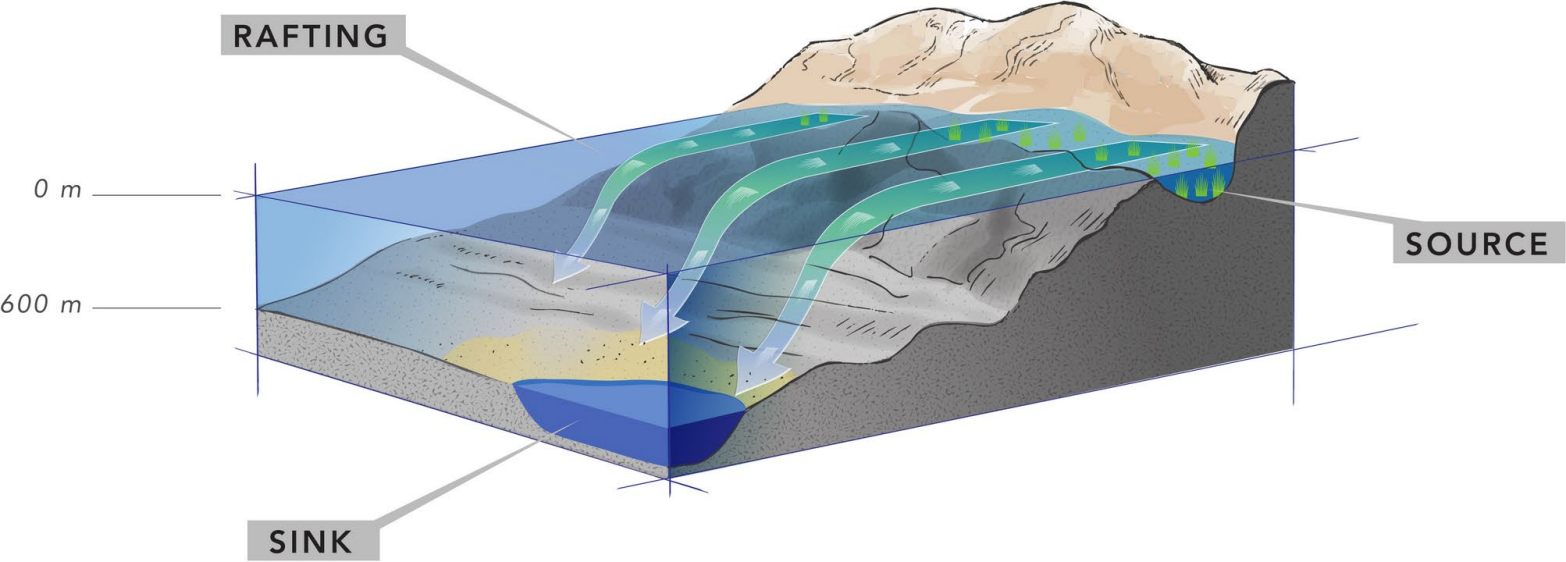
Schematic figure showing proposed mechanism of seagrass or other macrophyte rafting transport of large benthic foraminifera and other epiphytes, floating attached to the leaves and rhizomes before sinking to the seafloor.

These findings indicate that these foraminifera were transported via a mechanism other than sedimentation pathways typical for periplatform settings, such as gravity flows and density cascading^36^. Our observations suggest that seagrass and macroalgae rafting is the most likely mode of transport (Fig. 5). The simultaneous associated occurrence of LBF and seagrass remains in the Umluj brine pool, combined with observations of floating macrophytes far offshore, provides strong supporting evidence. Seagrass fragments can remain buoyant for multiple weeks^37–39^, enabling the transportation of their (facultatively) epiphytic communities across significant distances before eventually sinking to the seafloor^40–43^.

### Transport mechanisms of shallow-dwelling taxa to deep-sea sediments

**Fig. 6:**
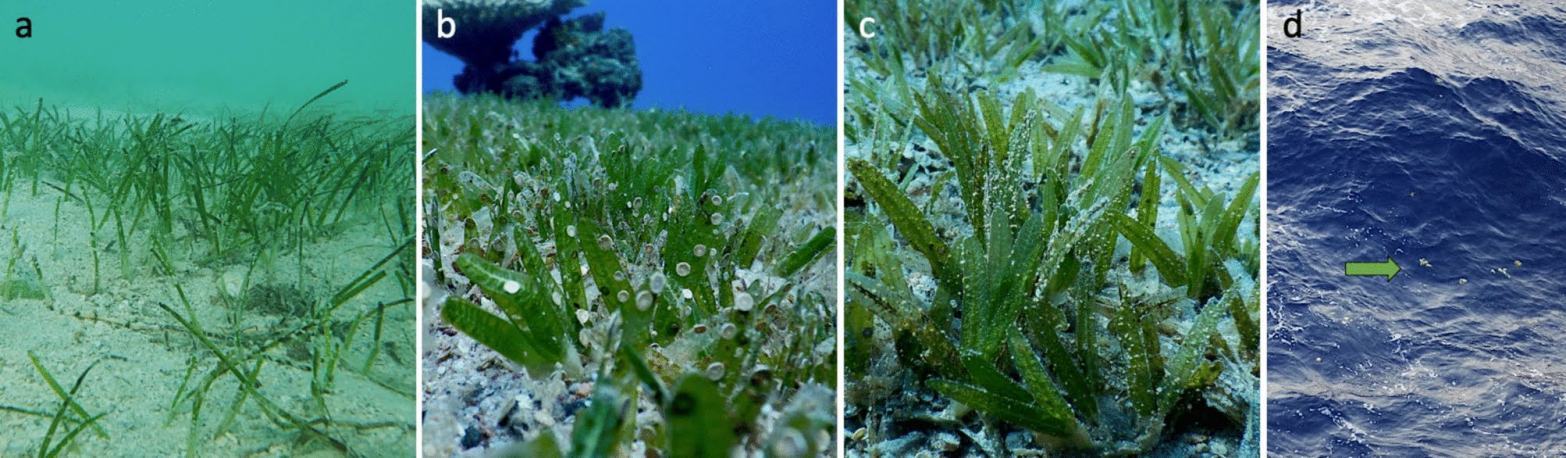
(**a**) Example of seagrass growing in the southern Al Wajh lagoon with epiphytes (photograph by FG); (**b**,**c**) close-ups of *Halophila* seagrass from Gulf of Aqaba featuring (facultatively) epiphytic foraminifera (**b**) mainly Soritidae in Eilat, Israel, and (**c**) Amphistegenidae in Dahab, Egypt; photographs by MS); (**d**) macrophytes observed floating along the RV Meteor during the M193 cruise some 25–50 km from any shallow coastal habitats.

The consistent appearance of LBF in deep-sea sediments suggests that they were transported offshore by rafting on seagrass and other macrophyte fragments (Fig. 5). The photosymbiont-bearing LBF species are well adapted to nutrient-poor, shallow-water euphotic habitats^1,44–46^, where they derive most of their energy from endosymbiotic microalgae residing within their tests^47,48^. LBF such as Soritidae and Amphisteginidae Cushman, 1927 are commonly found in back-reef lagoons and some of them live as facultative epiphytes on seagrass leaves and roots^49–57^ (Fig. 6b,c). While the distribution of small propagules via ocean currents is well-documented^58–60^, the transport of adult specimens over tens of kilometres without substantial physical damage requires a mechanism such as rafting. Similar reports of such direct ‚natural ‘ long-distance transport of shallow-water benthic foraminifera have so far been known from seagrass off the Banc d’Arguin, Mauritania^61^, the pelagic *Sargassum natans*^62^ and from debris items set adrift by a tsunami in the Pacific^63^. Most other reports, in comparison, of long-distance displacements of (invasive) foraminifera into distant habitats were commonly associated to human activities such as shipping through release of ballast water and sediments or from anchor mud (e.g., *Trochammina hadai* Uchio, 1962 and *Haynesina germanica* Ehrenberg, 1840)^64–66^.

Seagrass meadows along the Arabian Peninsula coastlines, including common species like *Halophila stipulacea* and *Thalassodendron ciliatum*, provide habitats for these epiphytic foraminifera (Fig. 6a). When seagrass fragments detach, they can float on the surface for considerable distances before sinking^40–43^. During the research cruise M-193, floating seagrass was observed at several locations (Fig. 6d), indicating ongoing processes of offshore transport (e.g., in the northern sector of the study area at 25–50 km offshore and from Al Wajh Bank). This observation is consistent with previous studies that documented the widespread occurrence of floating macrophytes as dispersal vectors in tropical marine ecosystems^39,41^. Like the seeds of many seagrass species, also their vegetative fragments can be dislodged and float over several weeks to months^37,39,67^. Especially the more delicate taxa like *Halophila* spp. (as in Fig. 6b,c), with their fine shallow-rooting rhizomes and small paddle-like leaves can spread rapidly through floating vegetative fragments that readily settle and establish new meadows^38,68,69^.

The more strongly abraded tests of *A. lessonii* and *A. radiata* found in the sample collected by the ROV (M193-71–1) have possibly undergone a different transport mechanism. Especially the latter species is commonly found in deeper habitats down to the mesophotic^2,15,48^, limiting the likelihood of rafting as transport means. The condition of the tests in addition indicates a less gentle downslope transport process such as off-platform shedding through gravitational transport where grain-to-grain contact leads to abrasion and breakage of delicate grains^2,70^. In this case, such coarse-grained material would be found directly downslope from the sourcing ecosystem. Occurrences of LBF transported from the shallow coastal habitats into deeper setting by high energy off-platform transport and rapid deposition are known from numerous fossil and modern carbonate depositional systems^71,72^. Given that the ROV collected the sediment containing those abraded foraminifer tests just at the bottom of a steep wall^19^, reaching from ~ 100 to 400 m water depth, we interpret them to have been winnowed off the platform by gravitational currents. In contrast, while transported attached to a seagrass blade, the test is floating on the surface, being thus protected from hydraulic energy, physical abrasion and other destructive forces.

### Evidence from the Umluj brine pool

The PARASOUND profile and steep shifts in physicochemical parameters at about 638 m in a depression south of Al Wajh Bank (stations M193-82 to M193-87; Fig. 2) confirmed the presence of the previously discovered Umluj brine pool^23^. The distinct pycnocline indicates minimal mixing with the overlying water owing to the high density of the brine (salinity > 180 PSU, density > 1146.1 kg/m^3^ and anoxic conditions below 646 m depth; though CTD values may be well out of instrumental and calibration ranges). These extreme conditions create a unique sedimentary environment, where degradation of fallen macrofauna and flora is hampered by the lack of oxidation and bioturbation processes, allow for the preservation of organic materials, such as the vegetative parts of seagrass as well as delicate foraminiferal tests.

The well-preserved macrophyte and foraminifera remains presented from Umluj brine pool strongly support the rafting hypothesis. The macrophyte fragments discovered in the pool include well-preserved leaves, rhizomes and stems of, e.g., *Halophila stipulacea* and *Thalassodendron ciliatum*, as well as brown algae (e.g., *Turbinaria ornata*), which are commonly found in shallow coastal and reef environments along the coast of Saudi Arabia^24,73,74^.

*H. stipulacea* is one of the most common species in the Red Sea where it flourishes in water depths between 1 and 20 m in lagoons, bays, and sheltered coastal areas, and recently is also spreading quickly throughout the eastern Mediterranean Sea^43,69,75,76^. *Th. ciliatum* is a common species on the eastern side of the Red Sea^77^, with an increasing abundance northward^78,79^, forming dense meadows at 0.5 to 15 m depth in areas with strong currents and wave action, for example near reefs and rocky coastlines. Likewise, *C. serrulata* preferably grows on sand or coral debris in the upper sublittoral zone, mainly between 1 and 10 m depth in sheltered bays and lagoons. The discovered macroalgae parts likely belong to *T. ornata* (Phaeophyta), *Sargassum* spp. and the rhodophyte *Laurencia* spp., all commonly found in shallow areas along the eastern coast of the Red Sea^80^.

These phytal substrates provide microhabitats (i.e., roots, rhizomes, stems and leaves) for diverse communities of temporarily and permanently attached epiphytic living benthic foraminifera^1,81–83^. For example, high densities of *A. lessonii*, *A. lobifera, S. orbiculus* and *A. hemprichii*, among others, live on *H. stipulacea* meadows in the Gulf of Aqaba^33,56,57,84^, *S. orbiculus* and *A. lobifera* may cover *Posidonia oceanica* leaves in the Mediterranean^81,85^, as well as macroalgae fronds, along with *A. hemprichii*^86^. Soritids and Amphistegenids were likewise found on algal mats around New Caledonia^82^ and *Thalassia* seagrass blades in Belize^55^. The discovery of pristine LBF, the specific combination of species, and their preservation status, therefore further support rafting as mode of transport and might imply that these macrophytes could act as vectors for dispersal over significant distances.

### Influence of local currents on seagrass rafting

The transport of seagrass flotsams in the Red Sea is largely influenced by the interaction of regional currents, wind patterns, and local hydrodynamics, where here the prevailing NNW wind system causes southerly flow of surface waters^87^. The distance at which LBF were found in deep-sea sediments suggests that the rafting distance of seagrass is limited, likely influenced by prevailing current patterns and convergence zones that confine the rafts within this range. The eastern boundary current, which flows northward along the coast of Saudi Arabia, coupled with the influence of mesoscale eddies and sub-gyres, likely plays a crucial role in directing the floating seagrass toward deeper offshore areas^6,8,27^. Convergence zones created by the interaction of these currents may act as temporary traps, allowing seagrass flotsams to accumulate and sink before travelling further offshore.

Further research into the specifics of this rafting process, including the factors influencing the distances covered and the eventual sinking of the flotsams, would enhance our understanding of its ecological implications. Moreover, oceanographic models might help clarify the pathways and distance limits of flotsams, which are critical to predicting how macrophyte rafting might influence sediment input and organism dispersal in offshore regions.

### Implications for sediment interpretation and biogeographic patterns

Rafting of shallow-water organisms to deep-sea environments has significant implications for interpreting the sedimentary record, but also for understanding marine biogeography and evolutionary patterns. Obviously, the presence of LBF in deep-sea sediments does not indicate local shallow-water conditions, but rather long-distance transport, potentially necessitating a re-evaluation of some paleoenvironmental reconstructions. This aligns with the work of Martorelli et al. (2011)^88^, who demonstrated that rafting could introduce allochthonous components into sedimentary records, altering interpretations of past marine conditions.

Rafting may also play a vital role in the biogeographic distribution of marine taxa, potentially aiding the spread of non-native taxa into new habitats, such as the current spreading of Lessepsian invaders throughout the Mediterranean. Regarding future tropicalization of the rapidly warming Mediterranean Sea^89,90^ the rapid invasion of *Amphistegina lobifera* and other LBF^33,91–98^ could hence be facilitated by their association with floating seagrasses like *H. stipulacea*. This species has also undergone fast range expansions in the eastern Mediterranean Sea and elsewhere^41,43,76,99^, and is expected to continue so^100,101^, with potentially massive impacts on associated ecosystem functions. Our case study corroborates the notion that such mechanisms may facilitate the co-dissemination and territorial expansion of marine species that live in association with each other, and could also contribute to the high dispersal capacity of some LBF^102^. Further research on the dynamics of these associations will provide insights into the processes that drive the distribution of marine species, to better anticipate changes in species ranges and implement effective management strategies to mitigate the impact of invasive species on native ecosystems.

### Further ecological implications of seagrass rafting

Active and passive dispersal is notoriously a key agent in the evolution and biogeography of terrestrial and marine organisms^41,42,67,103–105^, and this also applies to benthic foraminifera^58,59^. While the transport of epibionts on seagrass rafts can link different marine ecosystems, such as coastal seagrass meadows and deep-sea environments unidirectionally, it may also enhance the genetic connectivity between geographically separated populations bidirectionally in other settings where the environmental conditions between origin and depositional setting are more similar. Such connectivity may have cascading effects on ecosystem dynamics or contribute to gene flow^106^. While this is outside the scope of the current study, future research could target the potential of seagrass rafting in promoting ecosystem connectivity to, for example, further affect nutrient cycling or habitat structure across diverse marine environments. In our specific case study, rafting contributes to the offshore sedimentary record.

## Conclusions

This study provides compelling evidence for the passive rafting of shallow-dwelling LBF – and potentially other epiphytes – on seagrass and other macrophyte fragments from shallow coastal habitats into deep-sea sediments. It also underscores the potential for brine pool sediments to preserve detailed records of past marine conditions. Seagrass rafts might serve as important vectors for co-dispersal, allowing species to rapidly overcome long distances and establish new populations in remote locations. This could alter species distributions, especially under tropicalization scenarios that facilitate the spread of invasive species (e.g., *Halophila stipulacea* and *Amphistegina lobifera*)*,* which can outcompete native species and modify community dynamics, with consequences for biodiversity, ecosystem structure, and function^97,101^.

Our findings also highlight the importance of considering uncommon transport processes in paleoenvironmental reconstructions. Specifically, rafting must be distinguished from other transport mechanisms such as gravitational off-bank shedding because misidentification would affect the interpretation of sedimentary deposits. Otherwise, the presence of shallow-water (photic) organisms in deep water settings could be taken as evidence for near-surface conditions, while rather representing an “artifact” according to the dominant depositional context, introduced by floating macrophytes. The occurrence of well-preserved LBF in deep-water deposits presented here indicates this long-distance transport pathway. Once these organisms detach or sink with their substratum, they are not representative of the local sedimentary environment but instead indicate passive transport without reworking.

Further research into these transport mechanisms and their implications will likely enhance our understanding of marine biodiversity, ecosystem connectivity, and biogeographic patterns. At the end, such perspectives could even inform coastal management strategies by identifying key areas that facilitate connectivity and dispersal, thereby supporting marine biodiversity or ecosystem resilience.

## Supplementary Information

Supplementary Information.

## Data Availability

All sediment samples, oceanographic data collected along with core images and images and video footage from the ROV survey are stored at ZMT and can be made available upon reasonable request to H. Westphal. PARASOUND data is stored at the University of Hamburg and can be made accessible through T. Lüdmann.

## References

[1] Narayan, G. R. et al. Response of large benthic foraminifera to climate and local changes: Implications for future carbonate production. *Sedimentology* **69**, 121–161 (2022).

[2] Beavington-Penney, S. J. & Racey, A. Ecology of extant nummulitids and other larger benthic foraminifera: Applications in palaeoenvironmental analysis. *Earth Sci. Rev.* **67**, 219–265 (2004).

[3] Maillard, C. & Soliman, G. Hydrography of the Red Sea and exchanges with the Indian Ocean in summer. *Oceanol. Acta* **9**, 249–269 (1986).

[4] Jiang, H., Farrar, J. T., Beardsley, R. C., Chen, R. & Chen, C. Zonal surface wind jets across the Red Sea due to mountain gap forcing along both sides of the Red Sea. *Geophys. Res. Lett.* **36**, 1–6 (2009).

[5] Ehrmann, W., Wilson, P. A., Arz, H. W., Schulz, H. & Schmiedl, G. Monsoon-driven changes in Aeolian and fluvial sediment input to the central Red Sea recorded throughout the last 200 000 years. *Clim. Past* **20**, 37–52 (2024).

[6] Quadfasel, D. Red Sea circulation. *Ocean Curr.* 307 (2009).

[7] Eshel, G. & Naik, N. H. Climatological coastal jet collision, intermediate water formation, and the general circulation of the Red Sea. *J. Phys. Oceanogr.* **27**, 1233–1257 (1997).

[8] Zarokanellos, N. D., Papadopoulos, V. P., Sofianos, S. S. & Jones, B. H. Physical and biological characteristics of the winter-summer transition in the Central Red Sea. *J. Geophys. Res. Oceans* **122**, 6355–6370 (2017).

[9] Qurban, M. A. et al. In-situ observation of deep water corals in the northern Red Sea waters of Saudi Arabia. *Deep Sea Res. 1 Oceanogr. Res. Pap.* **89**, 35–43 (2014).

[10] Augustin, N. et al. The rifting to spreading transition in the Red Sea. *Earth Planet Sci. Lett.* **395**, 217–230 (2014).

[11] Petrovic, A. et al. Holocene sediment distribution in the Al Wajh platform lagoon (northern Red Sea, Saudi Arabia), a modern analogue for large rift basin carbonate platforms. *Sedimentology* **69**, 1365–1398 (2022).

[12] Feldens, P. & Mitchell, N. C. Salt Flows in the Central Red Sea. In *The Red Sea: The Formation, Morphology, Oceanography and Environment of a Young Ocean Basin* (eds Rasul, N. M. A. & Stewart, I. C. F.) 205–218 (Springer, 2015). 10.1007/978-3-662-45201-1_12.

[13] Orszag-Sperber, F., Harwood, G., Kendall, A. & Purser, B. H. A review of the evaporites of the Red Sea-Gulf of Suez rift. In *Sedimentation and Tectonics in Rift Basins Red Sea:- Gulf of Aden* (eds Purser, B. H. & Bosence, D. W. J.) 409–426 (Springer, 1998). 10.1007/978-94-011-4930-3_22.

[14] Roik, A., Röthig, T., Pogoreutz, C., Saderne, V. & Voolstra, C. R. Coral reef carbonate budgets and ecological drivers in the central Red Sea—A naturally high temperature and high total alkalinity environment. *Biogeosciences* **15**, 6277–6296 (2018).

[15] Bracchi, V. A. et al. Mesophotic foraminiferal-algal nodules play a role in the Red Sea carbonate budget. *Commun. Earth Environ.* **4**, 288 (2023).

[16] Ziegler, M., Roder, C., Bchel, C. & Voolstra, C. R. Niche acclimatization in Red Sea corals is dependent on flexibility of host-symbiont association. *Mar. Ecol. Prog. Ser.* **533**, 163–176 (2015).

[17] Degens, E. T. & Ross, D. A. *Hot Brines and Recent Heavy Metal Deposits in the Red Sea* (Springer, 1969).

[18] Taviani, M. Axial sedimentation of the Red Sea Transitional Region (22°–25° N): Pelagic, gravity flow and sapropel deposition during the late Quaternary. In *Sedimentation and Tectonics in Rift Basins Red Sea - Gulf of Aden* (eds Purser, B. H. & Bosence, D. W. J.) 467–478 (Springer, 1998). 10.1007/978-94-011-4930-3_25.

[19] Lüdmann, T. *et al. Red Sea Paleoenvironmental Evolution under Monsoon Fluctuations in the Pleistocene to Holocene - Cruise No. 193*. *METEOR-Berichte Red.*10.48433/cr_m193 (2024).

[20] Bosworth, W., Huchon, P. & McClay, K. The Red Sea and Gulf of Aden Basins. *J. Afr. Earth Sci.* **43**, 334–378 (2005).

[21] Mitchell, N. C., Ligi, M., Feldens, P. & Hübscher, C. Deformation of a young salt giant: Regional topography of the Red Sea Miocene evaporites. *Basin Res.* **29**, 352–369 (2017).

[22] Petrovic, A. et al. Fragmentation, rafting, and drowning of a carbonate platform margin in a rift-basin setting. *Geology* **51**, 242–246 (2023).

[23] Petrovic, A., Lakrani, P. & Vahrenkamp, V. Global to regional Holocene climate changes and events recorded in a near-slope deep sea brine-pool sediments of the NE Red Sea. EGU General Assembly 10.5194/egusphere-egu24-4630 (2024).

[24] Bruckner, A. *et al. Khaled Bin Sultan Living Oceans Foundation Atlas of Saudi Arabian Red Sea Marine Habitats*. (2012).

[25] Steinhauff, D. M., Abubshait, A. & Purkis, S. J. Red Sea Holocene carbonates: Windward platform margin and lagoon near Al-Wajh, northern Saudi Arabia. *J. Sedim. Res.* **91**, 847–875 (2021).

[26] Rowlands, G. et al. Satellite imaging coral reef resilience at regional scale. A case-study from Saudi Arabia. *Mar. Pollut. Bull.* **64**, 1222–1237 (2012). 22480935 10.1016/j.marpolbul.2012.03.003

[27] Aljohani, N. S. et al. Environmental impacts of thermal and brine dispersion using hydrodynamic modelling for Yanbu desalination plant, on the Eastern Coast of the Red Sea. *Sustainability (Switzerland)* **14**, 4389 (2022).

[28] Zhan, P. et al. Physical forcing of phytoplankton dynamics in the Al-Wajh lagoon (Red Sea). *Limnol. Oceanogr. Lett.* **7**, 373–384 (2022).

[29] Masetti, G., Smith, M. J., Mayer, L. A. & Kelley, J. G. W. Applications of the Gulf of Maine operational forecast system to enhance spatio-temporal oceanographic awareness for ocean mapping. *Front. Mar. Sci.* **6**, 1–16 (2020).

[30] Schlitzer, R. Ocean Data View. (2010).

[31] R Core Team. R: A language and environment for statistical computing. https://www.r-project.org/. (2016).

[32] Hottinger, L., Halicz, E. & Reiss, Z. *Recent Foraminiferida from the Gulf of Aqaba, Red Sea*. (1993).

[33] Merkado, G. et al. Molecular evidence for Lessepsian invasion of soritids (larger symbiont bearing benthic foraminifera). *PLoS One* **8**, e77725 (2013). 24204936 10.1371/journal.pone.0077725 PMC3812231

[34] Lee, J. J., Burnham, B. & Cevasco, M. E. A new modern soritid foraminifer, *Amphisorus saurensis* n. sp., from the Lizard Island Group (Great Barrier Reef, Australia). *Micropaleontology* **50**, 357–368 (2004).

[35] WoRMS Editorial Board. World Register of Marine Species. 10.14284/170. (2024).

[36] Wilson, P. A. & Roberts, H. H. Carbonate-periplatform sedimentation by density flows: A mechanism for rapid off-bank and vertical transport of shallow-water fines. *Geology* **20**, 713–716 (1992).

[37] Lai, S., Yaakub, S. M., Poh, T. S. M., Bouma, T. J. & Todd, P. A. Unlikely nomads: Settlement, establishment, and dislodgement processes of vegetative seagrass fragments. *Front. Plant Sci.* **9**, 1–11 (2018). 29491880 10.3389/fpls.2018.00160 PMC5817336

[38] Weatherall, E. J., Jackson, E. L., Hendry, R. A. & Campbell, M. L. Quantifying the dispersal potential of seagrass vegetative fragments: A comparison of multiple subtropical species. *Estuar. Coast. Shelf Sci.* **169**, 207–215 (2016).

[39] Wu, K., Chen, C. N. N. & Soong, K. Long distance dispersal potential of two seagrasses *Thalassia hemprichii* and *Halophila ovalis*. *PLoS One* **11**, 1–16 (2016). 10.1371/journal.pone.0156585 PMC4889049 27248695

[40] Duarte, C. M. & Krause-Jensen, D. Export from seagrass meadows contributes to marine carbon sequestration. *Front. Mar. Sci.*10.3389/fmars.2017.00013 (2017).

[41] Jahnke, M. et al. Patterns and mechanisms of dispersal in a keystone seagrass species. *Mar. Environ. Res.* **117**, 54–62 (2016). 27085058 10.1016/j.marenvres.2016.04.004

[42] Kendrick, G. A. et al. The central role of dispersal in the maintenance and persistence of seagrass populations. *Bioscience* **62**, 56–65 (2012).

[43] Thibaut, T. et al. Distribution of the seagrass *Halophila stipulacea*: A big jump to the northwestern Mediterranean Sea. *Aquat. Bot.* **176**, 8–11 (2022).

[44] BouDagher-Fadel, M. K. *Evolution and Geological Significance of Larger Benthic Foraminifera* (UCL Press, 2018). 10.14324/111.9781911576938.

[45] Hallock, P. Production of carbonate sediments by selected large benthic foraminifera on two Pacific coral reefs. *J. Sediment. Petrol.* **51**, 467–474 (1981).

[46] Hallock, P. Symbiont-bearing Foraminifera. In *Modern Foraminifera* (eds Sen Gupta, B. K.) 123–368 (Kluwer Academic Publishers, 1999).

[47] Lee, J. J. Algal symbiosis in larger foraminifera. *Symbiosis* **42**, 63–75 (2006).

[48] Prazeres, M. et al. Diversity and flexibility of algal symbiont community in globally distributed larger benthic foraminifera of the genus *Amphistegina*. *BMC Microbiol.* **21**, 1–16 (2021). 34488648 10.1186/s12866-021-02299-8 PMC8422653

[49] Buchan, O. C. *Relationships Between Large Benthic Foraminifera and Their Seagrass Habitats, San Salvador, Bahamas* (Auburn University, 2006).

[50] Fujita, K. & Hallock, P. A comparison of phytal substrate preferences of *Archaias angulatus* and *Sorites orbiculus* in mixed macroalgal-seagrass beds in Florida Bay. *J. Foraminifer. Res.* **29**, 143–151 (1999).

[51] Hohenegger, J., Yordanova, E., Nakano, Y. & Tatzreiter, F. Habitats of larger foraminifera on the upper reef slope of Sesoko Island, Okinawa, Japan. *Mar. Micropaleontol.* **36**, 109–168 (1999).

[52] Langer, M. R. & Hottinger, L. Biogeography of selected ‘larger’ foraminifera. *Micropaleontology* **46**, 105–126 (2000).

[53] Renema, W. Large benthic foraminifera from the deep photic zone of a mixed siliciclastic-carbonate shelf off East Kalimantan, Indonesia. *Mar. Micropaleontol.* **58**, 73–82 (2006).

[54] Renema, W. Terrestrial influence as a key driver of spatial variability in large benthic foraminiferal assemblage composition in the Central Indo-Pacific. *Earth Sci. Rev.* **177**, 514–544 (2018).

[55] Richardson, S. L. Seasonal variation in epiphytic foraminiferal biotas from *Thalassia* seagrass habitats, Twin Cays, Belize. *Atoll. Res. Bull.*10.5479/si.00775630.517.1 (2004).

[56] ter Kuile, B. & Erez, J. In situ growth rate experiments on the symbiont-bearing foraminifera *Amphistegina lobifera* and *Amphisorus hemprichii*. *J. Foraminifer. Res.* **14**, 262–276 (1984).

[57] Zohary, T., Reiss, Z. & Hottinger, L. Population dynamics of *Amphisorus hemprichii* (foraminifera) in the Gulf of Elat (Aqaba), Red Sea. *Eclogae Geologicae Helvetiae* **73**, 1071–1094 (1980).

[58] Alve, E. & Goldstein, S. T. Propagule transport as a key method of dispersal in benthic foraminifera (Protista). *Limnol. Oceanogr.* **48**, 2163–2170 (2003).

[59] Alve, E. & Goldstein, S. T. Dispersal, survival and delayed growth of benthic foraminiferal propagules. *J. Sea Res.* **63**, 36–51 (2010).

[60] Weinmann, A. E., Goldstein, S. T., Triantaphyllou, M. V. & Langer, M. R. Effects of sampling site, season, and substrate on foraminiferal assemblages grown from propagule banks from lagoon sediments of Corfu Island (Greece, Ionian Sea). *PLoS One* **14**, 1–27 (2019). 10.1371/journal.pone.0219015 PMC6599131 31251773

[61] Westphal, H. *et al. Phaeton - Paleoceanographic and Paleo-Climatic Record on the Mauritanian Shelf - Cruise No. MSM16/3 - October 13 - November 20, 2010 - Bremerhaven (Germany) - Mindelo (Cap Verde)*. (2014).

[62] Spindler, M. The pelagic gulfweed *Sargassum natans* as a habitat for the benthic Foraminifera *Planorbulina acervalis* and *Rosalina globularis*. *Neues Jahrbuch für Geologie und Paläontologie Monatshefte* **1980**, 569–580 (1980).

[63] Finger, K. L. Tsunami-generated rafting of foraminifera across the North Pacific Ocean. *Aquat. Invasions* **13**, 17–30 (2018).

[64] Eichler, P. P. B. et al. The occurrence of the invasive foraminifera *Trochammina hadai* Uchio in Flamengo Inlet, Ubatuba, São Paulo State, Brazil. *Micropaleontology* **64**, 391–402 (2018).

[65] McGann, M. & Sloan, D. Recent introduction of the foraminifer *Trochammina hadai* Uchio into San Francisco Bay, California, USA. *Mar. Micropaleontol.* **28**, 1–3 (1996).

[66] McGann, M., Ruiz, G. M., Hines, A. H. & Smith, G. A ship’s ballasting history as an indicator of foraminiferal invasion potential—an example from Prince William Sound, Alaska, USA. *J. Foraminifer. Res.* **49**, 434–455 (2019).

[67] McMahon, K. et al. The movement ecology of seagrasses. *Proc. R. Soc. B Biol. Sci.* **281**, 20140878 (2014). 10.1098/rspb.2014.0878 PMC4213608 25297859

[68] Smulders, F. O. H., Vonk, J. A., Engel, M. S. & Christianen, M. J. A. Expansion and fragment settlement of the non-native seagrass *Halophila stipulacea* in a Caribbean bay. *Mar. Biol. Res.* **13**, 967–974 (2017).

[69] Winters, G. et al. The tropical seagrass *Halophila stipulacea*: Reviewing what we know from its native and invasive habitats, alongside identifying knowledge gaps. *Front. Mar. Sci.* **7**, 1–28 (2020).32802822

[70] Fauquembergue, K. et al. Sedimentology of modern Bahamian carbonate slopes: Summary and update. *Geochem. Geophys. Geosyst.* **25**, e2023GC011077 (2024).

[71] Cotton, L. J. & Pearson, P. N. Cotton. Larger benthic foraminifera from the middle Eocene to Oligocene of Tanzania. *Austr. J. Earth Sci.* **105**, 189–199 (2012).

[72] Mateu-Vicens, G., Pomar, L. & Ferràndez-Cañadell, C. Nummulitic banks in the upper Lutetian ‘Buil level’, Ainsa Basin, South Central Pyrenean Zone: The impact of internal waves. *Sedimentology* **59**, 527–552 (2012).

[73] Al Harbi, S. M. Epiphytic microalgal dynamics and species composition on brown seaweeds (Phaeophyceae) on the northern coast of Jeddah, Saudi Arabia. *J. Oceanogr. Mar. Res.* **05**, 1–9 (2017).

[74] Ba-Akdah, M. A., Satheesh, S. & Al-Sofyani, A. A. Habitat preference and seasonal variability of epifaunal assemblages associated with macroalgal beds on the Central Red Sea coast, Saudi Arabia. *J. Mar. Biol. Assoc. U. K.* **96**, 1457–1467 (2016).

[75] Helber, S. B. et al. Nutrient history affects the response and resilience of the tropical seagrass *Halophila stipulacea* to further enrichment in its native habitat. *Front. Plant Sci.*10.3389/fpls.2021.678341 (2021). 34421939 10.3389/fpls.2021.678341 PMC8374242

[76] Wesselmann, M., Chefaoui, R. M., Marbà, N., Serrao, E. A. & Duarte, C. M. Warming threatens to propel the expansion of the exotic seagrass *Halophila stipulacea*. *Front. Mar. Sci.* **8**, 1–14 (2021).35685121

[77] Shaffai, A. El. *Field Guide of Seagrasses of the Red Sea*. (2011).

[78] Price, A. R. G. et al. Aspects of seagrass ecology along the eastern coast of the Red Sea. *Botanica Marina* **31**, 83–92 (1988).

[79] Green, E. P. & Short, F. T. *World Atlas of Seagrasses* (Published in association with UNEP-WCMC by the University of California Press, 2003).

[80] Ibraheem, I. B. M., Alharbi, R. M., Abdel-Raouf, N. & Al-Enazi, N. M. Contributions to the study of the marine algae inhabiting Umluj Seashore, Red Sea. *Beni Suef Univ. J. Basic Appl. Sci.* **3**, 278–285 (2014).

[81] Langer, M. R. Epiphytic foraminifera. *Mar. Micropaleontol.* **20**, 235–265 (1993).

[82] Debenay, J.-P. & Payri, C. E. Epiphytic foraminiferal assemblages on macroalgae in reefal environments of New Caledonia. *J. Foraminifer. Res.* **40**, 36–60 (2010).

[83] Mateu-Vicens, G., Khokhlova, A. & Sebastián-Pastor, T. Epiphytic foraminiferal indices as bioindicators in Mediterranean seagrass meadows. *J. Foraminifer. Res.* **44**(3), 325–339 (2014).

[84] Masawa, J. et al. A matter of choice: Understanding the interactions between epiphytic foraminifera and their seagrass host *Halophila stipulacea*. *Mar. Environ. Res.* **196**, 106437 (2024). 38479296 10.1016/j.marenvres.2024.106437

[85] Vohník, M. Bioerosion and fungal colonization of the invasive foraminiferan *Amphistegina lobifera* in a Mediterranean seagrass meadow. *Biogeosciences* **18**, 2777–2790 (2021).

[86] BadrElDin, A. M. & Hallock, P. M. Foraminifers associated with macroalgae on a wave-cut platform off Abu Qir coastal area, Egypt. *Egypt. J. Aquat. Res.* **48**, 389–395 (2022).

[87] Putri, I. et al. Architectural development of a land-attached carbonate platform in the African-Arabian Desert belt: The Late Pleistocene to Holocene evolution of the Al Wajh Platform, NE Red Sea, Saudi Araba. *J. Sedimentary Res.* **94**, 591–616 (2024).

[88] Martorelli, E. et al. Contourites offshore Pantelleria Island (Sicily Channel, Mediterranean Sea): Depositional, erosional and biogenic elements. *Geo-Mar. Lett.* **31**, 481–493 (2011).

[89] Bianchi, C. N. Biodiversity issues for the forthcoming tropical Mediterranean Sea. In *Biodiversity in Enclosed Seas and Artificial Marine Habitats* (eds Relini, G. & Ryland, J.) 7–21 (Springer, 2007).

[90] Albano, P. G. et al. The dawn of the tropical Atlantic invasion into the Mediterranean Sea. *Proc. Natl. Acad. Sci.* **121**, e2320687121 (2024). 38557179 10.1073/pnas.2320687121 PMC11009679

[91] Caruso, A. & Cosentino, C. The first colonization of the Genus *Amphistegina* and other exotic benthic foraminifera of the Pelagian Islands and south-eastern Sicily (central Mediterranean Sea). *Mar. Micropaleontol.* **111**, 38–52 (2014).

[92] Guastella, R. et al. “Hidden invaders” conquer the Sicily Channel and knock on the door of the Western Mediterranean Sea. *Estuar. Coast. Shelf Sci.* **225**, 106234 (2019).

[93] Hallock, P., Koukousioura, O. & Badreldin, A. M. Why *Amphistegina lobifera*, a tropical benthic foraminiferal species, is thriving at temperate latitudes in the Mediterranean Sea. *J. Foraminiferal Res.* **54**, 237–248 (2024).

[94] Langer, M. R., Weinmann, A. E., Lötters, S., Bernhard, J. M. & Rödder, D. Climate-driven range extension of *Amphistegina* (Protista, Foraminiferida): Models of current and predicted future ranges. *PLoS One* **8**, e54443 (2013). 23405081 10.1371/journal.pone.0054443 PMC3566174

[95] Schmidt, C. et al. Recent invasion of the symbiont-bearing foraminifera *Pararotalia* into the eastern Mediterranean facilitated by the ongoing warming trend. *PLoS One* **10**, e0132917 (2015). 26270964 10.1371/journal.pone.0132917 PMC4536047

[96] Stulpinaite, R., Hyams-Kaphzan, O. & Langer, M. R. Alien and cryptogenic Foraminifera in the Mediterranean Sea: A revision of taxa as part of the EU 2020 Marine Strategy Framework Directive. *Mediterr. Mar. Sci.* **21**, 719–758 (2020).

[97] Weinmann, A. E., Koukousioura, O., Triantaphyllou, M. V. & Langer, M. R. Invasive shallow-water foraminifera impacts local biodiversity mostly at densities above 20%: The case of Corfu Island. *Web Ecol.* **23**, 71–86 (2023).

[98] Yokes, M. B., Meric, E. & Avsar, N. On the presence of alien foraminifera *Amphistegina lobifera* Larsen on the coasts of the Maltese Islands. *Aquat. Invasions* **2**, 439–441 (2007).

[99] Telesca, L. et al. Seagrass meadows (*Posidonia oceanica*) distribution and trajectories of change. *Sci. Rep.* **5**, 1–14 (2015). 10.1038/srep12505 PMC4516961 26216526

[100] Beca-Carretero, P., Teichberg, M., Winters, G., Procaccini, G. & Reuter, H. Projected rapid habitat expansion of tropical seagrass species in the Mediterranean Sea as climate change progresses. *Front. Plant Sci.* **11**, 1–15 (2020). 33304358 10.3389/fpls.2020.555376 PMC7701102

[101] Beca-Carretero, P. et al. Climate change and the presence of invasive species will threaten the persistence of the Mediterranean seagrass community. *Sci. Total Environ.* **910**, 168675 (2024). 37981144 10.1016/j.scitotenv.2023.168675

[102] Prazeres, M. et al. High dispersal capacity and biogeographic breaks shape the genetic diversity of a globally-distributed reef-dwelling calcifier. *Ecol. Evol.*10.1002/ece3.6335 (2020). 32607205 10.1002/ece3.6335 PMC7319125

[103] Kinlan, B. P. & Gaines, S. D. Propagule dispersal in marine and terrestrial environments: A community perspective. *Ecology* **84**, 2007–2020 (2003).

[104] Kokko, H. & López-Sepulcre, A. From individual dispersal to species ranges: Perspectives for a changing world. *Science* **1979**(313), 789–791 (2006). 10.1126/science.1128566 16902127

[105] Croteau, E. K. Causes and consequences of dispersal in plants and animals. *Nat. Educ. Knowl.* **3**, 12 (2010).

[106] Govaert, L. et al. Integrating fundamental processes to understand eco-evolutionary community dynamics and patterns. *Funct. Ecol.* **35**, 2138–2155 (2021).

