# Supplementary material for: Seagrass-rafted large benthic foraminifera transported into the deep Red Sea
Source: Sci Rep. 2025 Feb 17;15:5724. doi: 10.1038/s41598-025-90047-7 (PMC11833106; doi:10.1038/s41598-025-90047-7)
Supplement: Supplementary file 1 — Supplementary Information. [file 41598_2025_90047_MOESM1_ESM.docx]

**Supplementary Material**

**Seagrass-rafted large benthic foraminifera transported into the deep Red Sea,** Scientific Reports, 2025, https://doi.org/10.1038/s41598-025-90047-7

Marleen Stuhr^1,9,*^, Hildegard Westphal^1,2,3,9^, Fabio Marchese^4^, Guillem Mateu-Vicens^5^, Francesca Giovenzana^4^, Thomas Lüdmann^6^, Volker Vahrenkamp^3^, Marco Taviani^7,8^

^1^Leibniz Centre for Tropical Marine Research (ZMT), Bremen, Germany. ^2^Department of Geosciences, Bremen

University, Bremen, Germany. ^3^Physical Sciences and Engineering Division, King Abdullah University of Science

and Technology (KAUST), Thuwal, Saudi Arabia. ^4^Biological and Environmental Sciences and Engineering Division,

King Abdullah University of Science and Technology (KAUST), Thuwal, Saudi Arabia. ^5^Department of Biology,

Universitat de Les Illes Balears, 07122 Palma de Mallorca, Spain. ^6^Institute of Geology, University of Hamburg,

Hamburg, Germany. ^7^ISMAR-CNR, Bologna, Italy. ^8^Stazione Zoologica ‘Anton Dohrn’, Napoli, Italy. ^9^Marleen Stuhr

and Hildegard Westphal contributed equally to this work. *email: [marleen.stuhr@leibniz-zmt.de](mailto:marleen.stuhr@leibniz-zmt.de)

**
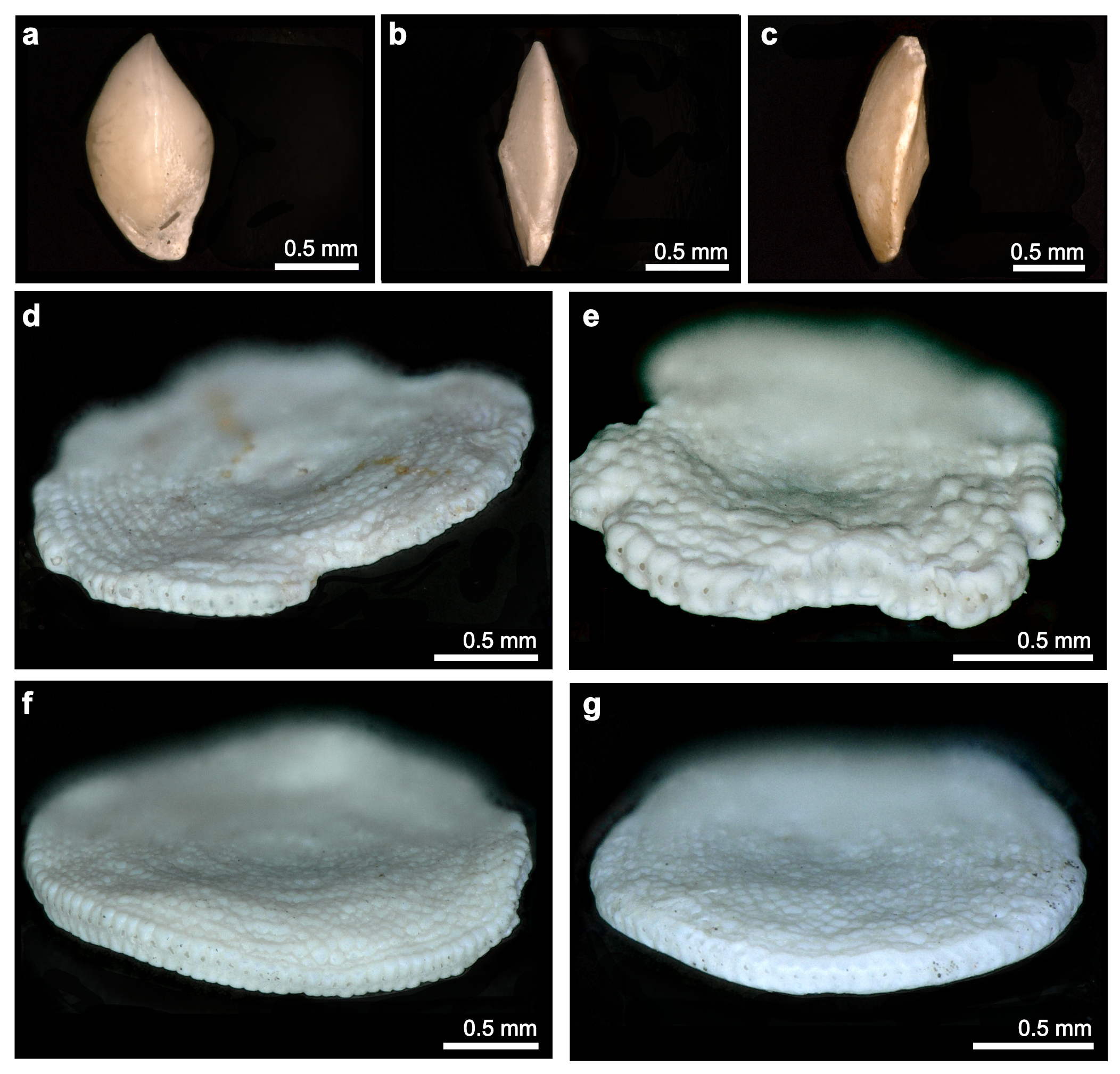
**

**Figure S1.** Lateral views of a) *Amphistegina lobifera*, station 11; b) *A. radiata*, station 71; c) *A. lessonii*, station 71; d) *Sorites variabilis*, station 12; e) *Amphisorus hemprichii*, station 73; f) *Sorites orbiculus*, station 11, and g) station 16, referring to a-d, f, h, and k in Fig. 3.
